# Comparison of renal impairment post-myocardial infarction with reduced and preserved left ventricular function in rats with normal renal function

**DOI:** 10.1080/0886022X.2020.1752241

**Published:** 2020-04-25

**Authors:** Zhuzhi Wen, Zun Mai, Xiaolin Zhu, Yangxin Chen, Dengfeng Geng, Jingfeng Wang

**Affiliations:** a Department of Cardiology, Sun Yat-sen Memorial Hospital, Sun Yat-sen University, Guangzhou, China; b Breast Tumor Center, Sun Yat-sen Memorial Hospital, Sun Yat-sen University, Guangzhou, China

**Keywords:** Cardiac dysfunction, myocardial infarction, rennin angiotensin system, renal impairment, podocytes

## Abstract

This study aimed to compare the renal impairments in post-myocardial infarction (MI) rats with normal renal biochemical parameters at baseline with versus without cardiac dysfunction and explore the potential mechanisms involved in these differences. Sprague–Dawley rats with permanent ligation of coronary artery were used as MI models. Renal function, histological and molecular changes were compared between the reduced ejection fraction (EF) (EF < 40%) group and the preserved EF (EF ≥ 40%) group 3 or 9 weeks post-MI. The results revealed that blood cystatin C increased significantly at 9 but not 3 weeks, but it was not associated with cardiac dysfunction. Renal fibrosis and inflammatory cell infiltrations increased significantly in the reduced EF group than in the preserved EF group at 3 and 9 weeks. Glomerular podocyte injury, identified by increased immunostaining for desmin and decreased immunostaining for Wilms’ tumor-1, was more significant in the reduced EF group than in the preserved EF group at 9, but not 3 weeks. The number of p16^ink4a^-positive and 8-hydroxy-2′-deoxyguanosine-positive podocytes was greater in the reduced EF group than in the preserved EF group at both time points. These changes were associated with increased expression of angiotensin II type 1/2 receptors at both time points. In conclusion, our study demonstrated that cardiac dysfunction accounted for substantially severity in renal parenchymal impairment in a partially time-dependent manner, and local activation of angiotensin II receptors, increased cell senescence and oxidative stress, and enhanced inflammatory reaction may be potential modulators participated in the deterioration of renal parenchymal injury.

## Introduction

Left ventricular dysfunction following acute myocardial infarction (MI) is a leading cause of mortality and morbidity throughout the world. Deterioration of renal function occurs commonly and will further increase mortality in post-MI subjects with left ventricular dysfunction, particularly in patients with preexisting renal impairment [1,2]. Therefore, it is important to explore the underlying mechanisms involved in renal tissue damage with the goal of providing effective strategies to prevent the progression of renal impairment post-MI with subsequent left ventricular dysfunction.

Our previous study [3] and another report [4] have revealed the presence of injury to the podocytes, key parenchymal cells within the glomerular, in rat models with left ventricular dysfunction. Our prior research suggests that undue activation of the local renin angiotensin system (RAS) within the kidneys is an important marker for the pathogenesis of podocyte injury post-MI [3]. The inhibition of RAS with angiotensin II type 1 receptor (AT-1R) blockers could prevent the initiation and deterioration of podocyte injury, providing new insight into the treatment of renal impairment post-MI with resultant left ventricular dysfunction [3,4].

Renal parenchymal injury is closely associated with the deterioration of renal function following MI [5]. Until now, research on renal impairment has been limited to functional index of biochemical changes in MI with subsequent heart failure. Although increased renal injury post-MI dysfunction has been previously reported, little has been known about renal parenchymal impairment after MI with or without left ventricular dysfunction, especially in those with normal biochemical changes in renal function. Therefore, the present study aimed to determine differences in renal impairment, especially in podocyte injury, in post-MI rats with normal baseline renal functional biochemical parameters with or without reduced left ventricular function, and to further explore the potential mechanisms involved in these differences.

## Materials and methods

### Experimental MI and treatments

Experimental MI was induced in rats by left coronary artery ligation as described in our previous work [3,6,7]. Briefly, male Sprague-Dawley rats weighing about 300 g were anesthetized with sodium pentobarbital (intraperitoneal, 30 mg/kg), intubated, and then ventilated with a rodent respirator. A left thoracotomy was performed via the left fourth intercostal space and the heart was exposed. After the pericardium was opened, the left anterior descending coronary artery was ligated with a 6-0 silk suture before the chest was closed with a soft tube in the cavity in order to allow air and blood to escape. After ventilation with room air for approximately 5 min, the animal was gradually weaned from the respirator once spontaneous respiration had resumed and observed until completely conscious.

**Figure 1.**
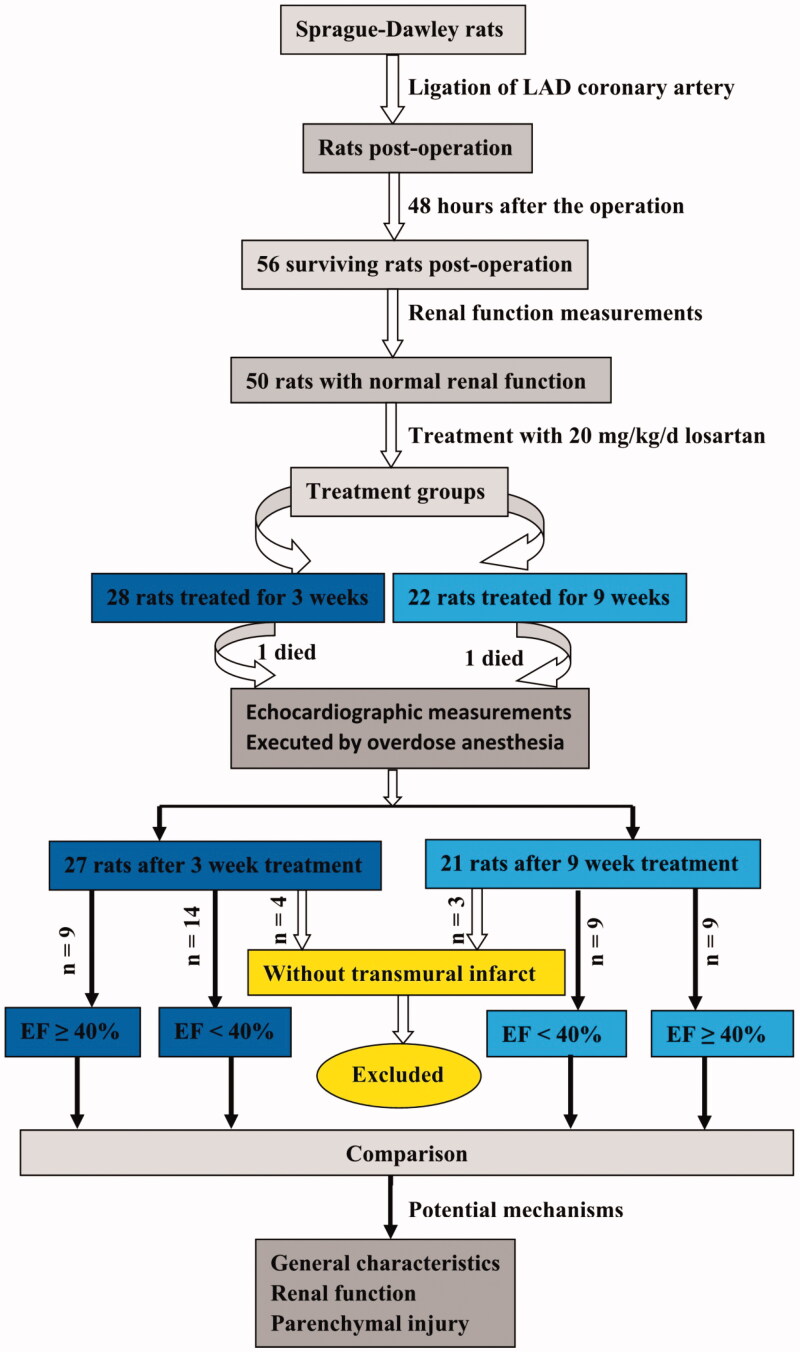
Schematic description of the study design.

Forty-eight hours after surgery, animals surviving MI that presented normal serum creatinine levels (53–96 μmol/L, mean 76 μmol/L) were treated with losartan (20 mg/kg/d) via gastric gavage daily for 3 or 9 weeks. MI rats were further divided into the reduced ejection fraction (EF) group (EF < 40%, *n* = 14 and 9 for 3 and 9 weeks, respectively) and the preserved EF group (EF ≥ 40%, *n* = 9 and 9 for 3 and 9 weeks, respectively). At the end of each time point, the differences in general characteristics, biochemical changes of renal function and renal parenchymal injuries were compared between groups, and potential mechanisms involved in these differences were also explored (Figure 1).

### Ethics statement

All animals in the present study were obtained from the Laboratory Animal Center of Sun Yat-sen University (Guangzhou, China). All animal procedures performed were in accordance with the ethical standards of the institution (Institutional Animal Care and Use Committee, Sun Yat-Sen University, SYSU-IACUC-2019-B106).

### Echocardiographic and blood pressure measurements

The parameters of left ventricular function and anatomic changes, as well as blood pressure, were obtained as previously described [3,6,7]. Echocardiographic measurements were performed 3 and 9 weeks post-treatment using a high-resolution echocardiographic imaging system equipped with a 16 MHz transducer (Vevo2100, Visualsonics, Canada). The rats were anesthetized with 3% isoflurane mixed with oxygen. A two-dimensional short-axis view of the left ventricle was obtained at the midpapillary level for the recording of M-mode tracings. Three consecutive measurements of the systolic and diastolic blood pressure, as well as heart rate were taken 48 h post-MI and during the 3, 6 and 9-week treatment period in conscious rats using a tail-cuff plethysmograph (model BP-98A; Softron Co., Japan).

### Biochemical examinations in experimental animals

Biochemical examinations were performed using the methods as described in our previous studies [3,6,7]. Trunk or tail vessel blood was sampled at different time points (48 h, 3 weeks and 9 weeks post-MI) for biochemical assessment. Serum creatinine, blood urea nitrogen, and glucose were detected using commercial kits (Beijing Leadman Biochemistry Technology Co. Ltd, China), according to the manufacturers’ instructions. Urine samples of 24 h were collected from metabolic cages at the 3 and 9 weeks post-MI, centrifuged and the supernatants were subjected to measurement of total urinary protein by colorimetric assays using a protein assay kit (Beijing Leadman Biochemistry Technology Co. Ltd, Beijing, China).

### Histological and immunohistochemical examinations

After either a 3- or 9-week treatment period, heart and kidney tissues were obtained for histological and immunohistochemical assessments according to the previously described protocols [3,6,7]. Immediately after anesthesia with an overdose of sodium pentobarbital (100 mg/kg, i.p.), hearts and kidneys were quickly obtained and weighed. Kidney and heart tissues were cut and fixed in 4% formalin and embedded in paraffin for histological and immunohistochemical assays. Kidney and heart sections (4 μm thickness) were stained with hematoxylin and eosin for routine histological examination, and with Masson’s trichrome reagents for assessment of renal fibrosis.

The slides were de-paraffinized in xylene, rehydrated through graded alcohol, immersed in 3% hydrogen peroxide to block endogenous peroxidase activity, and antigen-retrieved by pressure cooking in citrate buffer (pH = 6). After blocking for nonspecific binding, the slides were incubated with anti-Wilms’ tumor-1 (WT-1) (Santa Cruz, CA), anti-desmin (Dako, Denmark), anti-p16^ink4a^ (Santa Cruz, CA), anti-8-hydroxy-2′-deoxyguanosine (8-OHdG) (Japan Institute for the control of Aging, Japan), anti-AT-1R (Abcam, Cambridge, MA), and anti-AT-2R (Abcam, Cambridge, MA) antibodies overnight at 4 °C. The slides were then incubated with a secondary antibody (Dako, Denmark) and stained with 3,3-diaminobenzidine. Finally, the sections were counterstained with Mayer’s hematoxylin, dehydrated, and mounted. A negative control was obtained by replacing the primary antibody with a normal murine IgG. The percentage of desmin-positive area within each glomerular and the number of WT-1, p16^ink4a^ and 8-OHdG-positive podocytes per glomerulus were counted by a pathological expert in a blind manner using Image-Pro Plus software.

### Enzyme-linked immunosorbent assay

Serum samples collected at 3 and 9 weeks post-MI were tested for content of cystatin C and insulin-like growth factor-1 (IGF-1). Enzyme-linked immunosorbent assays were completed using commercial kits against IGF-1 (MG100, R&D Systems) and cystatin C (MSCTC0, R&D Systems) according to the manufacturer’s instructions.

### Statistical analysis

All quantitative data are presented as the mean ± SD and were compared by *t* test or ANOVA with LSD *post hoc* test within subgroups. Two-tailed *p* values < 0.05 were considered significant. All statistical analyses were performed with the software package SPSS 19.0 (IBM, USA) for Windows.

## Results

### General characteristics

**Figure 2.**
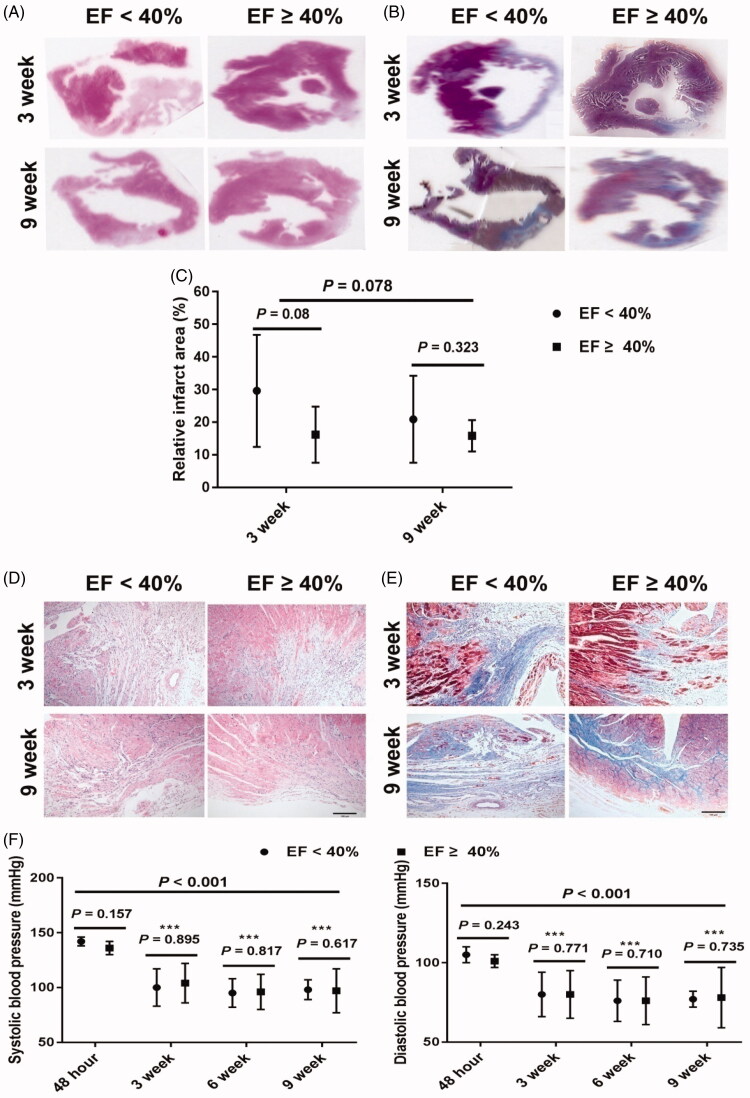
Representative pathologic changes of infarct heart and changes of blood pressure after losartan treatment in rats post-MI. (A) Representative ventricular sections of MI by hematoxylin and eosin staining at 3 and 9 weeks (original magnification, ×6). (B) Representative ventricular sections of MI by Masson’s trichrome staining at 3 and 9 weeks (original magnification, ×6). (C) Relative infarct area based on the data from hematoxylin and eosin staining and Masson’s trichrome staining at 3 and 9 weeks. (D) Representative changes of cardiac myocytes in infarct border zone by hematoxylin and eosin staining at 3 and 9 weeks (original magnification, ×200). (E) Representative changes of cardiac fibrosis in infarct border zone by Masson’s trichrome staining at 3 and 9 weeks (original magnification, ×200). (F) Changes of blood pressure 48 h post-MI and 3, 6 and 9 weeks after losartan treatment post-MI, ***p < 0.001 vs. 48 h timepoint. MI: myocardial infarction; EF: ejection fraction.

Overall, we found that left ventricular function, as demonstrated by EF and fractional shortening, was further improved at 9 weeks mark than at 3 weeks mark, suggesting long term treatment with losartan would further increase effects (Table 1). Additionally, for rats with EF < 40%, EF and fraction shortening was also improved at 9 weeks, when compared with that at 3 weeks. Furthermore, we also observed increases in the left ventricular systolic diameter, left ventricular diastolic diameter, left ventricular systolic volume and left ventricular diastolic volume at 9 weeks in rats with EF < 40% compared to rats with EF ≥ 40%. However, at 3 weeks only the left ventricular systolic diameter and left ventricular systolic volume increased. It was observed that rats with EF < 40% from both 3 and 9 weeks had a larger infarct area than rats with EF ≥ 40% from both 3 and 9 weeks (*p* = 0.003), but there was no significant difference of infarct area between rats with EF < 40% and EF ≥ 40% at each time point (Figure 2(A–C)). The sample number may account for these differences. Furthermore, there was no significant difference of infarct area between the two time points overall and in either EF group. When compared to rats with EF ≥ 40%, rats in EF < 40% groups had less viable myocardium, much more inflammatory cells and cardiac fibrosis in infarct border zone at both 3 and 9 weeks (Figure 2(D,E)). The arrangement of myocardium in infarct border zone in EF ≥ 40% groups was better than the EF < 40% groups at each time point. There was no significant difference in blood pressure and glucose levels observed between the two groups at the two time points (Table 1). In order to observe the changes of blood pressure induced by losartan treatment, the blood pressure values of 13 rats (6 in EF < 40% group and 7 in EF ≥ 40% group, respectively) in 9-week group were measured 48 h post-MI and at 3, 6 and 9 weeks after MI treatment with losartan. Our findings revealed that there were significant differences in both systolic and diastolic blood pressure among the four time points (Figure 2(F)). Rats treated with losartan at either time point had significantly lower blood pressure when compared to the blood pressure at 48 h post-MI. However, there was no significant difference in blood pressure between rats with reduced and preserved EF during the 3, 6 and 9-week treatment period, respectively.

**Table 1.** General characteristics and biological parameters in rats post-myocardial infarction.

	3 weeks		9 weeks		*p*^†^
	EF < 40%	EF ≥ 40%	EF < 40%	EF ≥ 40%	
Number (*n*)	14	9	9	9	
Body weight (g)	332.1 ± 42.5	320.0 ± 12.3	335.7 ± 18.0	351.74 ± 19.6**^&^**	0.139
Heart weight (g)	0.82 ± 0.08	0.81 ± 0.15	0.80 ± 0.07	0.79 ± 0.04	0.377
Heart weight/body weight (×10^−3^)	2.48 ± 0.20	2.54 ± 0.54	2.37 ± 0.19	2.25 ± 0.09	0.025
Heart rate (beat/min)	390 ± 53	365 ± 51	338 ± 47*	322 ± 29	<0.001
Systolic blood pressure (mmHg)	99 ± 13	104 ± 17	98 ± 13	97 ± 11	0.548
Diastolic blood pressure (mmHg)	76 ± 12	84 ± 16	78 ± 9	75 ± 9	0.704
LVsD (mm)	7.39 ± 1.12	5.87 ± 0.78**^#^**	7.97 ± 0.81	6.14 ± 0.50**^‡^**	0.874
LVdD (mm)	8.58 ± 1.08	8.23 ± 1.00	9.56 ± 0.85*	8.53 ± 0.57**^‡^**	0.080
LVsV (µL)	297.4 ± 97.9	176.4 ± 52.4**^#^**	344.2 ± 75.8	191.9 ± 37.2**^‡^**	0.944
LVdV (µL)	410.7 ± 111.3	372.5 ± 96.3	515.1 ± 98.6*	400.5 ± 59.5**^‡^**	0.087
Ejection fraction (%)	28.6 ± 6.1	52.8 ± 6.27**^#^**	33.4 ± 4.7*	53.1 ± 7.2**^‡^**	0.043
Fractional shortening (%)	14.0 ± 3.2	28.4 ± 4.01**^#^**	16.8 ± 2.6*	28.7 ± 4.8**^‡^**	0.045
Blood glucose (mmol/L)	5.57 ± 0.98	6.00 ± 0.75	5.61 ± 1.42	5.52 ± 1.36	0.763

Data are presented as means ± SD. *p* value based on *t* test. *p*^†^ indicates the statistics for all rats between the two time points. ^#^*p* < 0.05 for EF < 40% vs. EF ≥ 40% at 3 weeks; **^‡^***p* < 0.05 for EF < 40% vs. EF ≥ 40% at 9 weeks; *p < 0.05 for EF < 40% between 3 and 9 weeks; **^&^***p* < 0.05 for EF ≥ 40% between 3 and 9 weeks.

EF: ejection fraction; LVsD: left ventricular systolic diameter; LVdD: Left ventricular diastolic diameter; LVsV: left ventricular systolic volume; LVdV: left ventricular diastolic volume.

### Changes in renal functional parameters

At 3 weeks, the renal weight and the ratio of renal weight to body weight were significantly higher in rats with EF < 40% compared to rats with EF ≥ 40% (Table 2). We did not observe a significant difference in serum creatinine, blood urea nitrogen, and urine protein between the rats with EF < 40% and EF ≥ 40% at 3 and 9 weeks. Overall, post-MI rats at 9 weeks had significantly higher levels of cystatin C than those at 3 weeks, but there was no significant difference in cystatin C levels between rats in the reduced EF and preserved EF groups at either time point.

**Table 2.** Biological characteristics of renal parameters in rats post-myocardial infarction.

	3 weeks		9 weeks		*p*^†^
	EF < 40%	EF ≥ 40%	EF < 40%	EF ≥ 40%	
Number (*n*)	14	9	9	9	
Renal weight (g)	2.16 ± 0.41	1.92 ± 0.06**^#^**	1.91 ± 0.20	1.91 ± 0.25	0.071
Renal weight/body weight (×10^−3^)	6.48 ± 0.73	6.00 ± 0.24**^#^**	5.69 ± 0.43*	5.44 ± 0.59**^&^**	<0.001
Renal/Heart weight ratios	1.31 ± 0.14	1.22 ± 0.19	1.20 ± 0.10	1.20 ± 0.13	0.074
Urine protein (mg/d)	0.45 ± 0.34	0.52 ± 0.24	1.00 ± 0.75	0.84 ± 0.76	0.119
Serum creatinine (μmol/L)	71.2 ± 18.6	69.8 ± 5.53	72.2 ± 7.6	72.8 ± 8.0	0.723
Blood urea nitrogen ( mmol/L )	6.74 ± 2.46	6.38 ± 0.88	6.30 ± 1.69	6.20 ± 1.56	0.566
Blood cystatin C (mg/L)	2.65 ± 0.36	2.81 ± 0.22	3.10 ± 0.56*	3.25 ± 0.46**^&^**	0.002

Data are presented as means ± SD. *p* value based on t-test. *p*^†^ indicates the statistics for all rats between the two time points. ^#^*p* < 0.05 for EF < 40% vs. EF ≥ 40% at 3 weeks; *p < 0.05 for EF < 40% between 3 and 9 weeks; **^&^***p* < 0.05 for EF ≥ 40% between 3 and 9 weeks.

EF: ejection fraction.

### Renal histological changes

**Figure 3.**
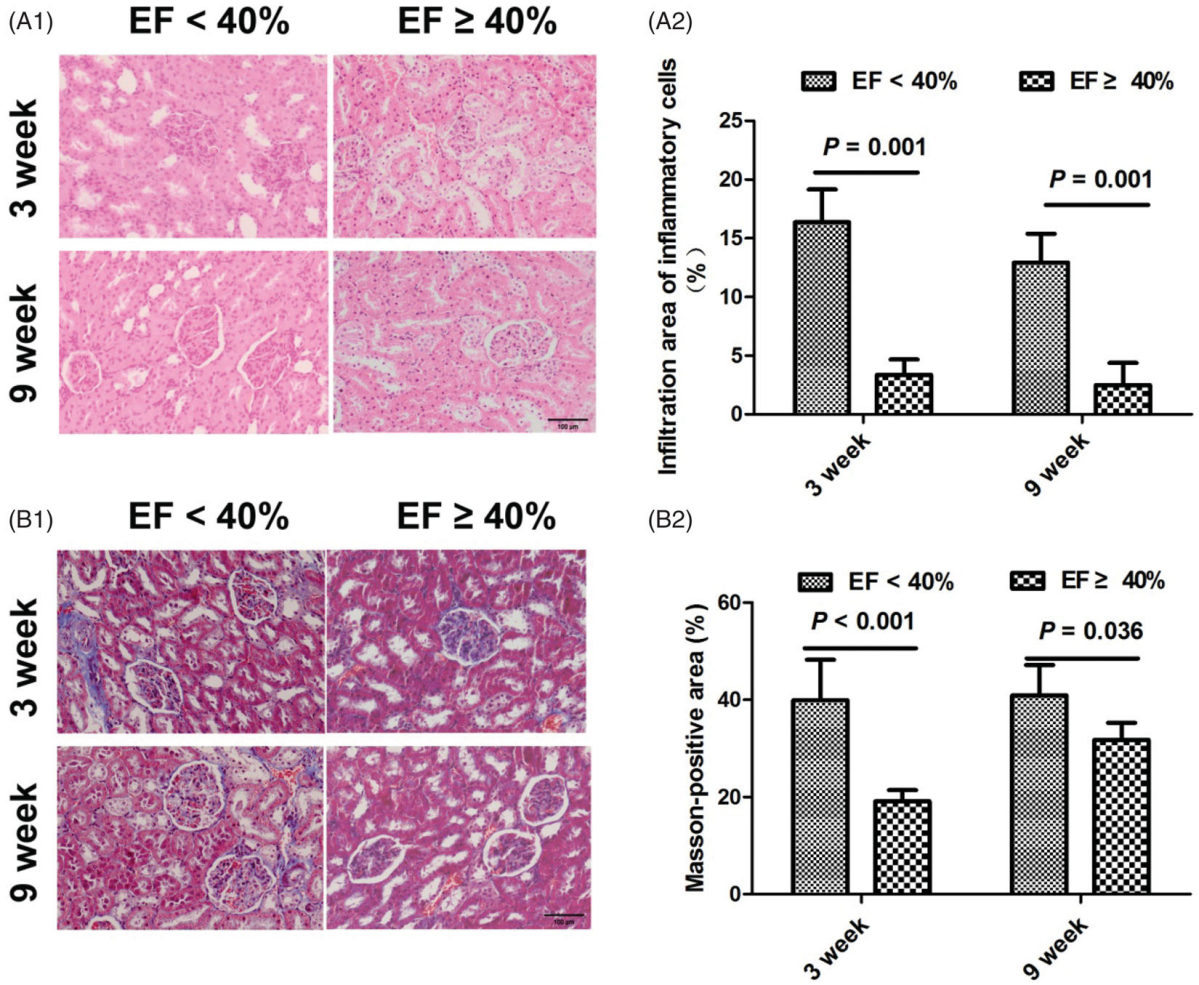
Representative inflammatory cell infiltration by hematoxylin and eosin staining and Masson’s trichrome staining suggesting renal fibrosis (blue staining) in MI rats at 3 and 9 weeks (original magnification, ×200). (A1) MI induced inflammatory cell infiltration within and surrounding the renal glomerulus at 3 and 9 weeks. (A2) The relative percentage of infiltration area of inflammatory cells after adjustment for normal control at 3 and 9 weeks. (B1) MI induced renal fibrosis within and surrounding the glomerulus at 3 and 9 weeks. (B2) The relative Masson-positive area suggesting renal fibrosis at 3 and 9 weeks. MI: myocardial infarction; EF: ejection fraction.

The prevalence of infiltration of inflammatory cells was demonstrated within and surrounding the renal glomerulus in animals with reduced EF at both time points (Figure 3(A1,A2)). However, there was no significant difference in the infiltration of inflammatory cells between the two time points in each group. Masson’s trichrome staining demonstrated significant renal fibrosis within and surrounding the renal glomerulus in animals with EF < 40% when compared with animals with EF ≥ 40% at both time points (Figure 3(B1,B2)). Despite this, renal fibrosis of the renal glomerulus was not significant between 3 and 9 weeks in either group.

### Glomerular podocyte changes

**Figure 4.**
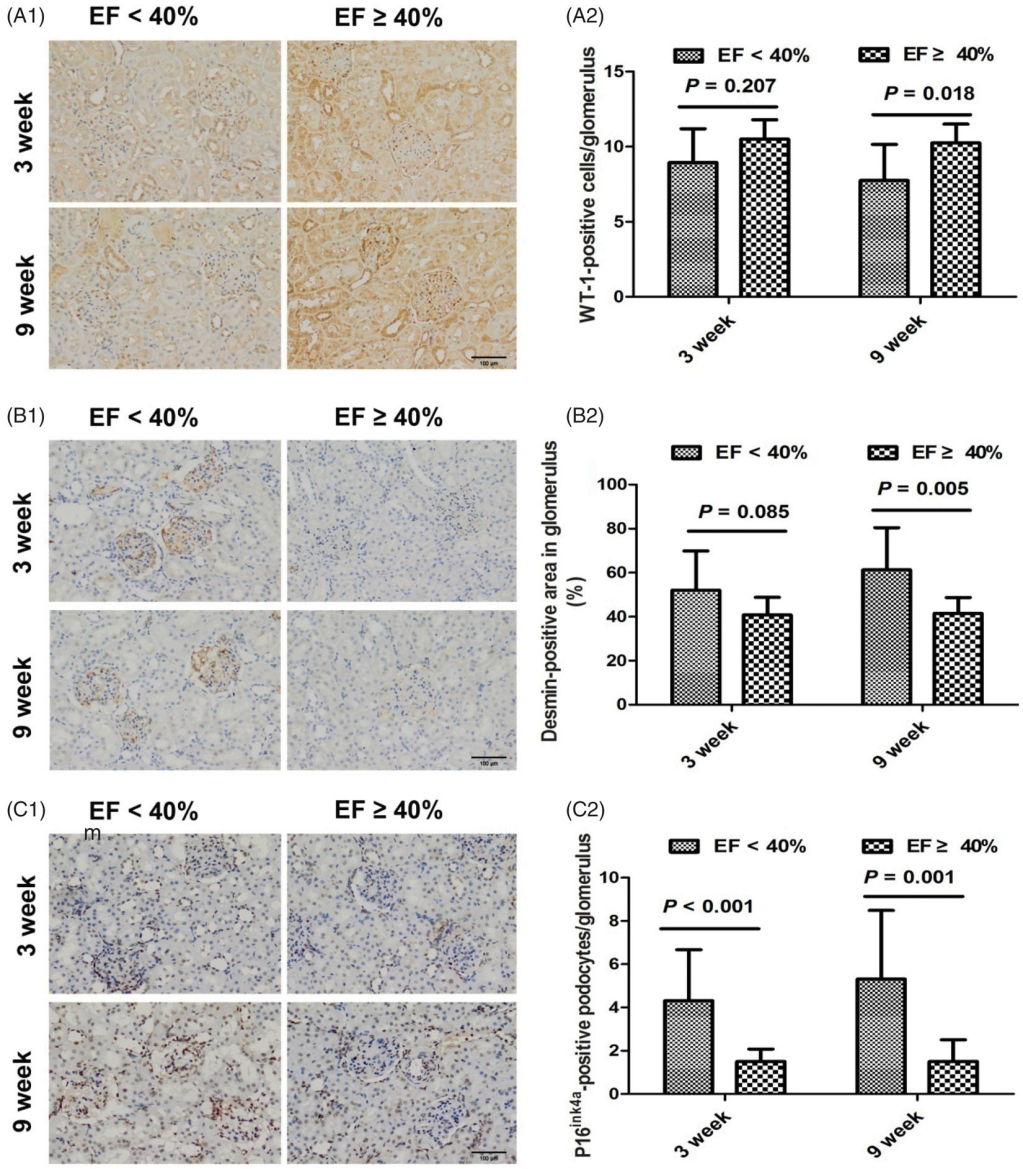
Representative podocyte injury in myocardial infarction rats at 3 and 9 weeks. (A1) Immunohistochemical staining for WT-1-positive podocytes at 3 and 9 weeks. (A2) The average number of WT-1-positive podocytes per glomerulus at 3 and 9 weeks. (B1) Immunohistochemical staining for desmin at 3 and 9 weeks. (B2) The relative percentage of desmin-stained area of total glomerular area in the glomerulus at 3 and 9 weeks. (C1) Immunohistochemical staining for p16^ink4a^ at 3 and 9 weeks. (C2) The average number of p16^ink4a^-positive podocytes per glomerulus at 3 and 9 weeks. EF: ejection fraction; WT-1: Wilms’ tumor-1; original magnification, ×200.

When compared with MI animals with EF ≥ 40%, MI animals with EF < 40% had a greater number of injured podocytes, as identified by increased desmin-positive immunostaining, but a decreased number of podocytes in the glomerulus overall, as identified by WT-1-positive immunostaining (both *p* < 0.01). Interestingly, we found significant differences in desmin-positive and WT-1-positive podocytes between the two groups at 9 weeks, but not at 3 weeks (Figure 4(A1–B2)). As tested by the p16^ink4a^ assay, the number of senescent podocytes in the glomerulus in EF < 40% animals was significantly higher than in EF ≥ 40% animals overall (*p* < 0.001) and at both the 3 and 9 weeks time points (Figure 4(C1,C2)). However, the changes in podocytes identified by desmin, WT-1 and p16^ink4a^ were not significant between the two time points.

### Potential mechanisms involved in MI-induced renal impairment

**Figure 5.**
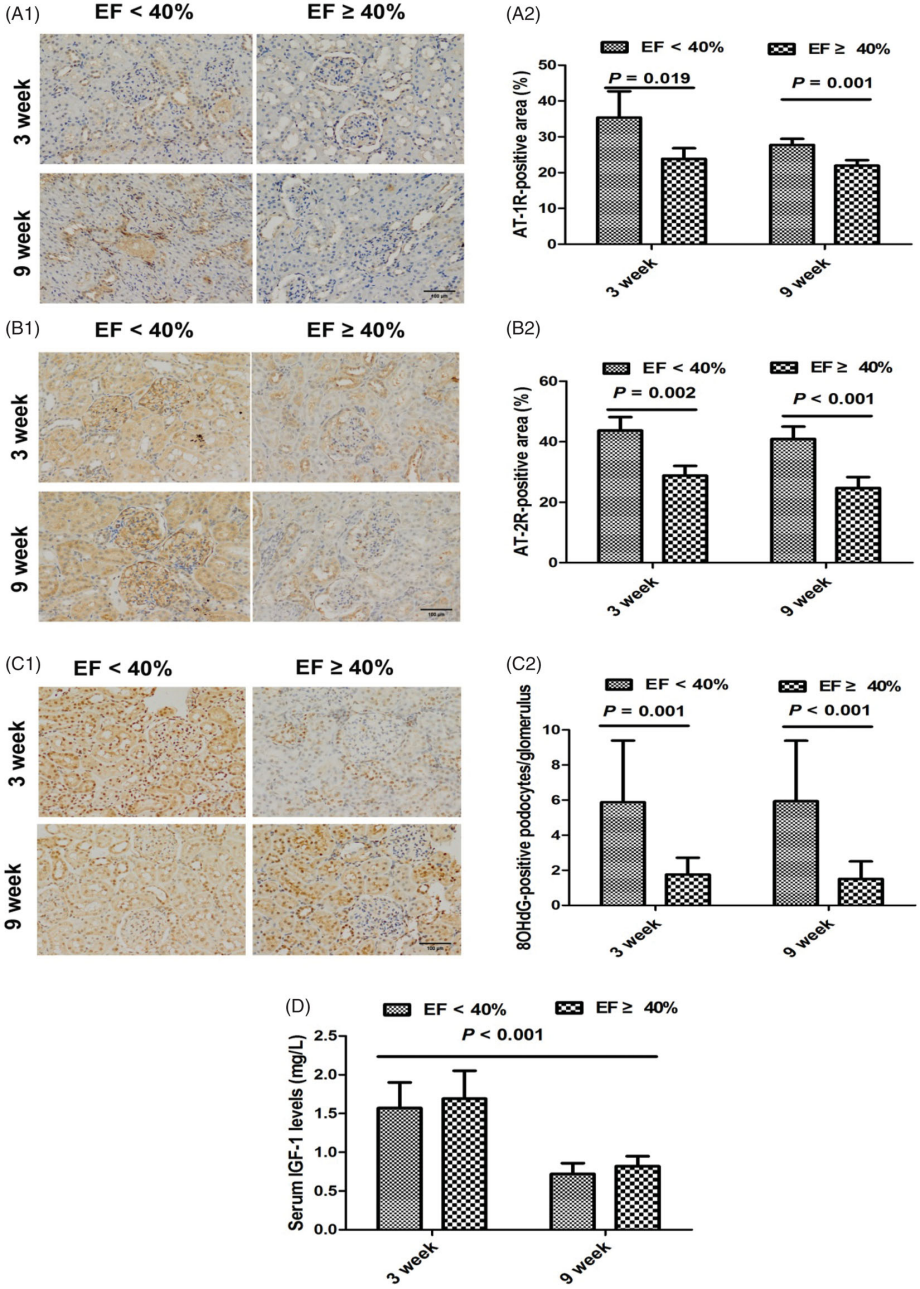
Potential modulators involved in renal injury in myocardial infarction rats at 3 and 9 weeks. (A1) Immunohistochemical staining for AT-1R at 3 and 9 weeks. (A2) The relative percentage of AT-1R-stained area within and surrounding the glomerulus at 3 and 9 weeks. (B1) Immunohistochemical staining for AT-2R at 3 and 9 weeks. (B2) The relative percentage of AT-2R-stained area within and surrounding the glomerulus at 3 and 9 weeks. (C1) Immunohistochemical staining for 8-OHdG-positive podocytes at 3 and 9 weeks. (C2) The average number of 8-OHdG-positive podocytes per glomerulus at 3 and 9 weeks. (D) Serum insulin-like growth factor-1 (IGF-1) levels at 3 and 9 weeks. AT-1/2R: angiotensin II type 1/2 receptor; 8-OHdG: 8-hydroxy-2′-deoxyguanosine; EF: ejection fraction; original magnification, ×200.

There was a significant increase in the proteins of AT-1R and AT-2R in renal cortical tissue in EF < 40% animals, as compared with those with EF ≥ 40%, at both time points (Figure 5(A1–B2)). However, AT-1R and AT-2R expression did not differ significantly between the time points in either group.

We examined 8-OHdG, a marker of oxidative stress, by immunostaining of the glomerular podocytes, and found that rats with reduced EF had higher levels of 8-OHdG in the glomerulus when compared to the EF ≥40% group overall (*p* < 0.001) and at both 3 and 9 weeks (Figure 5(C1,C2)). We did not observe a significant difference in the 8-OHdG-positive podocytes between 3 and 9 weeks. There was also no significant difference in 8-OHdG-positive podocytes between 3 and 9 weeks in either group. Our results indicated that MI rats at 9 weeks had lower levels of serum IGF-1 than those at 3 weeks. Moreover, we found there was an obvious difference in the IGF-1 levels between 3 and 9 weeks in both two groups, although there was no statistical significance in the difference in IGF-1 levels between the two groups at both time points (Figure 5(D)).

## Discussion

Cardiorenal syndrome is now a frequently recognized pathological phenomenon, and the combined dysfunction of the heart and kidneys increases morbidity and mortality. The impact of cardiac dysfunction further deteriorates the impairment of renal function and parenchymal damage post-MI [8]. The most critical predictor of acute kidney injury in MI patients is heart failure rather than other risk factors, including contrast medium volume, baseline renal function, diabetes and age [9]. Post-MI patients with cardiac dysfunction present more often with acute kidney injury throughout their hospitalization [10]. Other findings also suggest that cardiac dysfunction post-MI contributes to accelerated intrinsic kidney injury in chronic kidney disease [11].

Despite these important findings, there is a void of in-depth studies comparing renal parenchymal impairment after MI with and without reduced left ventricular function, especially in those with normal biochemical parameters of renal function. It has been demonstrated that rats with MI showed only mildly accelerated glomerular remodeling and microalbuminuria, with little change in renal hemodynamics and immunity [12]. However, cardiac dysfunction occurring post-MI would significantly accelerate glomerular remodeling and podocyte injury, and thereby alter local hemodynamics and immunity. Our present study indicated that MI rats with reduced EF had significant and serious podocyte damage in the glomerular, which was identified by decreased glomerular staining of the podocyte marker, WT-1, and increased glomerular staining of the marker of podocyte injury, desmin. Rats with reduced EF also exhibited enhanced upregulation of p16^ink4a^, a key mediator of cell cycle inhibition for stress and senescence induced by aberrant signaling, in glomerular podocytes. Our study also demonstrated that blood cystatin C, rather than blood creatinine and blood urea nitrogen, may be a preferential marker for the early renal impairment that occurs post-MI. We also found a significant association of increased cystatin C levels and a higher hazard for hospitalization due to heart failure, as well as an independent association with higher mortality in heart failure [13,14]. Moreover, podocyte injury, as identified by increased desmin-positive and decreased WT-1-positive staining, was significantly different at 9 weeks, rather than at 3 weeks post-MI, which was accompanied by a significant increase in cystatin C at 9 weeks but not 3 weeks following MI. This finding suggested that not only left ventricular dysfunction but also the duration post-MI was associated with the early pathological renal damage in those with normal serum creatinine levels.

The pathophysiology in cardiorenal syndrome is poorly understood and likely involves interrelated concepts such as low cardiac output, increased venous pressure and reduced renal perfusion, neurohormonal and inflammatory activation, and local changes [15–17]. There are several possible inflammatory mechanisms that underlie the renal damage following cardiac dysfunction in rat MI models, which may include tubular cell apoptosis, macrophage infiltration and interstitial fibrosis [18]. Our current study revealed the significant occurrence of increased inflammatory cell infiltration and fibrosis within the kidneys in post-MI rats with reduced EF. Other findings also revealed a significant increase in interstitial fibrosis in the renal cortex in post-MI rats with less severe renal dysfunction [19]. This study, along with our previous findings [3,6], lends further support to the concept that MI-induced activation of the local RAS is essentially involved in the onset and progression of renal injury. Activation of renal AT-1R contributes to the pathogenesis of progressive renal injury in rats with surgically induced MI [20]. The findings revealed that proteinuria was associated with infiltration of mononuclear cells and increased AT-1R protein that is primarily expressed in these cells. However, renal injuries have still been demonstrated in MI rats in spite of losarten treatment, especially in those with reduced EF. Furthermore, our results, in combination with other’s [21], suggested that the beneficial effects of angiotensin II receptor blockers on renal injury are also owed to increasing angiotensin II effects transduced through the AT-2R pathway.

Our findings further demonstrated that podocyte injury and senescence are related to an upregulation in the oxidative stress marker of 8-OHdG in rats after MI, especially in those with reduced EF. Oxidative stress leads to most deleterious consequences in the renal cortex, while antioxidant treatment can protect against renal damage post-MI [19]. Our previous study revealed that local expression of IGF-1/IGF-1 receptor signaling decreased significantly within the kidneys post-MI, and that losartan might restore the expression of these abnormalities [3]. Inhibition of glomerular oxidative stress and upregulation of IGF-1 may be important modulators involved in the process of podocyte protection in post-MI cardiac dysfunction. The changes in serum IGF-1 observed in our current study also suggested that IGF-1 might be a potential modulator of the post-MI duration, but not the changes of left ventricular function on renal injury.

In conclusion, post-MI rats with left ventricular dysfunction suffered from substantially more severe renal impairment, especially over a long period of time following MI. Renal fibrosis and podocyte damage were significantly indicated as important manifestations. Local activation of angiotensin II receptors, increased oxidative stress, and enhanced inflammatory reaction may be potential modulators contributing to the renal parenchymal injury that occurs post-MI. We observed significant podocyte injuries at 9 weeks, rather than at 3 weeks, and blood cystatin C and serum IGF-1 displayed the reverse changes at 9 weeks post-MI, suggesting that the long-term decrease in the level of serum IGF-1 might account for the increased renal impairment post-MI, regardless of left ventricular dysfunction. The pathological changes resulted from renal injury post-MI observed in the present study may bring clinicians insight into the mechanisms and modulators involved in cardiorenal syndrome. However, renal damage and the potential mechanisms that drive it were not tested in genetically modified animal models with relevant pharmacological agents, both of which are important limitations of the present study. The surgical MI models are not equivalent to atheroscherotic ones in the clinical settings and the underlying pathology in MI rats do not quite fit in with humans. Measuring certain markers does also not mean to determine potential mechanisms involved in MI-induced renal impairment. Furthermore, the changes of blood pressure after using losartan during 3 and 9-week treatment period might be important regarding cardiac function, renal function and inflammation. Therefore, further research using gene modified animal models and relevant pharmacological agents may help further elucidate the related mechanisms.
